# Editorial: Immunosenescence and Immunoexhaustion in Chronic Kidney Disease and Renal Transplantation

**DOI:** 10.3389/fmed.2022.874581

**Published:** 2022-04-05

**Authors:** Maria J. Stangou, Asimina Fylaktou, Milena Ivanova Ivanova-Shivarova, Ioannis Theodorou

**Affiliations:** ^1^Department of Nephrology, Aristotle University of Thessaloniki, Thessaloniki, Greece; ^2^Department of Immunology, National Peripheral Histocompatibility Center, Hippokration Hospital, Thessaloniki, Greece; ^3^Department of Clinical Immunology, University Hospital Aleksandrovska, Medical University, Sofia, Bulgaria; ^4^Assistance Publique Hopitaux De Paris, Paris, France

**Keywords:** renal function, immunosenescence and exhaustion, kidney transplantation, gut microbiome, glomerulonephritis, hemodialysis

The Immune System (IS) and Kidney function are closely and interactively connected (1). Dysregulation of the IS, as it occurs in systemic autoimmune diseases, may affect kidneys through several pathways, including immune complex deposition, signaling transduction pathways or complement activation, leading to an excessive variety of glomerular and interstitial disorders (2, 3). Moreover, chronic kidney disease (CKD) *per se*, characterized as an “inflamm-aging” condition, seems to affect immune integrity, in a way similar to aging process; mainly directed to adaptive immunity, leading to a shift of lymphocytes toward senescent and exhausted phenotypes (4, 5). Clinical consequences, including increased cardiovascular risk, susceptibility to infections and reduced response to immunization, are critical (6, 7). Following kidney transplantation (KT), renal function is reinstated; yet initiation of immunosuppressive treatment may simply change the scene of IS disturbances.

Apparently, the interaction between IS and kidneys is multifaceted and extremely important in many aspects (8). This special issue aimed to gather participation of investigators to present their latest findings upon immune-senescent and exhaustion phenomena relative to renal function. A group of eminent authors participated, with nine papers, four original research articles, four reviews, and one case report.

The gut microbiome may act as a bridge between kidneys, aging and IS disorders (9). Microbiota evolves normally from newborn to elderly, as a result of epigenetic mechanisms, environmental factors, personal habits, nutrition, etc. Recent studies have implicated microbiota in the pathogenesis of primary glomerular diseases, such as IgAN and membranous nephropathy (8). In this issue, the review from Stavropoulou et al., describes that CKD side-effects, such as metabolic disorders, dyslipidemia, oxidative stress, converging to gut dysbiosis, may generate the “inflammaging” status, leading to immune senescent alterations.

Predicting the progression of a glomerular disease is a useful tool for clinicians in order to determine treatment approach. Papasotiriou et al., presented the ability of survival models, recently designed to predict long term outcome of IgA Nephropathy (IgAN), the most common glomerulonephritis worldwide and common cause of end stage renal disease (ESRD). The authors showed that predictive models may overestimate the risk probability, however they were accurate to distinguish high risk IgAN patients.

As the adaptive immunity is predominantly affected in CKD, resulting in lymphocyte phenotypic changes, Duni et al., analyzed specific lymphocyte subsets, including B cells, CD14++CD16+ monocytes, Natural Killer cells (NKs) and regulatory T lymphocytes (Tregs), in peritoneal dialysis (PD) patients. B cell lymphopenia, together with increased CD14++CD16+ monocytes and NK cells were closely associated with the presence of CVD, but also there was a direct association of these lymphocyte subtypes with adequacy of PD method and fluid balance.

Apart from the increased incidence of cardiovascular disease, CKD patients have a phenotype of premature aging, characterized by frailty, muscle wasting, and osteoporosis. The shift toward senescent T cell subtypes, as the result of a premature thymic involution, is compensated by homeostatic expansion of highly differentiated memory T lymphocytes (10). Potential therapeutic interventions to prevent or even reverse ESRD-related premature immune-senescence are described by Ducloux et al., and include increased physical activity and dietary interventions to modulate gut microbiota and reduce levels of protein bound uremic toxins (p-cresol, p-cresyl sulfate). Administration of growth hormone is a safe procedure, which leads to increased IGF1 levels and may reverse thymic involution. The use of median cut-off or vitamin E-coded dialyzers are preferred in hemodialysis, although PD seems more advantageous. KT, although essentially restores renal function, it cannot reverse thymus involution and its effect on recovering adaptive immunity is still under investigation.

Increased risk of infection in ESRD has become more profound the last 2 years, during the COVID-19 pandemic. Co-morbid conditions, such as CKD and organ transplantation, are associated with the highest mortality risk from COVID-19 infection. Betjes presents pathogenic mechanisms of severe COVID-19 infection in CKD patients and the role of immune senescence in the evolution of the disease. The contracted TcR repertoire in naïve T lymphocytes and reduced numbers of plasmocytoid dendritic cells, in CKD patients, may have a profound negative effect on control of viral infections. Moreover, CKD and elderly patients are characterized by increased proportion of CD4+CD28 null cells, advanced differentiated cells, highly activated and poorly controlled, responding with a cytokine storm, responsible for lung parenchyma damage.

In the study of Weiger et al., response to BNT162b2 vaccination was significantly lower in HD patients compared to healthy controls. After the first dose almost 70% of patients were seronegative. Anti-spike IgG levels were increased only after the second dose, and almost disappeared over the following 4 months. These findings describe a late response to vaccination in HD patients, with limited duration, and they advocate for the specific management of these patients during COVID-19 pandemic with reinforced vaccination schedules.

KT is undoubtedly the treatment of choice for CKD, although this usually comes after protracted periods undergoing on dialysis. Patients are usually exposed to foreign HLAs, which are processed into smaller peptides loaded onto HLA class II and expressed on antigen presenting cells. After recognition of the above T cell epitopes (TEs), naïve CD4+ T lymphocytes are differentiated into donor-reactive memory cells, responsible for the early de novo DSA (dnDSA) formation after transplantation. Tomosugi et al., proved that evaluation of shared TEs by the *in silico* assay using the PIRCHE-II algorithm, can estimate donor-reactive memory CD4+ T cells and predict the risk of early dnDSA formation after KT.

Phenotypic markers of lymphocytes (CD3, CD4, CD8, CD19, CD56) monocytes (CD14, CD16, CD86, and CD54), and endothelium-derived microvesicles (MV) (annexin V+CD31+CD41–) were estimated in KT patients and compared to healthy controls and CKD patients, by Ceprian et al. Surprisingly, B-cell lymphopenia together with the increased numbers of T-cytotoxic lymphocytes and activated monocytes persisted after KT, and correlated negatively with MVs. Findings of the present study were novel and may explain the persistent adverse outcome of CVD despite KT.

B-cell lymphopenia and acquired agammaglobulinemia further deteriorates after reaching ESRD and is complicated by recurrent infections, however, as Pavlakou et al., presented that administration of IVIG may be curative and can safely be continued even after KT, together with immunosuppression.

We believe the present issue will be a valuable implement to promote further investigation in order to understand the Immune System-Kidney axis.

## Author Contributions

MS: conceptualization and writing the original draft. AF, MI-S, and IT: conceptualization, writing—review, and editing. All authors approved the submitted version.

## Conflict of Interest

The authors declare that the research was conducted in the absence of any commercial or financial relationships that could be construed as a potential conflict of interest.

## Publisher's Note

All claims expressed in this article are solely those of the authors and do not necessarily represent those of their affiliated organizations, or those of the publisher, the editors and the reviewers. Any product that may be evaluated in this article, or claim that may be made by its manufacturer, is not guaranteed or endorsed by the publisher.

## References

[1] Lioulios G Fylaktou A Papagianni A Stangou M. T cell markers recount the course of immunosenescence in healthy individuals and chronic kidney disease. Clin Immunol. (2021) 225:108685. 10.1016/j.clim.2021.108685 33549833

[2] Bantis K Stangou M Kalpakidis S Hatziadamou M Daikidou DV Lioulios G. Systemic complement activation in anti-neutrophil cytoplasmic antibody-associated vasculitis and necrotizing glomerulonephritis. Nephrology (Carlton). (2021) 26:30–7. 10.1111/nep.13747 32602136

[3] Bellur SS Roberts ISD Troyanov S. Reproducibility of the Oxford classification of immunoglobulin A nephropathy, impact of biopsy scoring on treatment allocation and clinical relevance of disagreements: Evidence from the VALidation of IGA study cohort. Neph Dial Transplant. (2019) 34:1681–90. 10.1093/ndt/gfy337 30561721

[4] Sampani E Stangou M Daikidou DV Nikolaidou V Asouchidou D Dimitriadis C. Influence of end stage renal disease on CD28 expression and T-cell immunity. Nephrology (Carlton). (2021) 26:185–96. 10.1111/nep.13784 32935413

[5] Sampani E Daikidou DV Lioulios G Xochelli A Mitsoglou Z Nikolaidou V. CD28null and regulatory T cells are substantially disrupted in patients with end-stage renal disease due to diabetes mellitus. Int J Mol Sci. (2021) 22:2975. 10.3390/ijms22062975 33804135 PMC8001943

[6] Lioulios G Stangou M Sarafidis PA Tsouchnikas I Minasidis I Vainas A. Chronic therapy with sucroferric oxyhydroxide does not affect iron and anemia markers in dialysis patients. Blood Purif. (2020) 49:440–7. 10.1159/000505432 32050202

[7] Ducloux D. ESRD-associated immune phenotype depends on dialysis modality and iron status: clinical implications. Immun Ageing. (2018) 15:16. 10.1186/s12979-018-0121-z 30026783 PMC6050655

[8] Ivanova M Creary LE Al Hadra B Lukanov T Mazzocco M Sacchi N. 17th IHIW component “Immunogenetics of Ageing” - New NGS data. Hum Immunol. (2019) 80:703–13. 10.1016/j.humimm.2019.07.287 31331679 PMC6773488

[9] Rodrigues FG Ormanji MS Heilberg IP Bakker SJL de Borst MH. Interplay between gut microbiota, bone health and vascular calcification in chronic kidney disease. Eur J Clin Invest. (2021) 51:e13588. 10.1111/eci.13588 33948936 PMC8459296

[10] Betjes MGH Langerak AW Klepper M Litjens NHR. A very low thymus function identifies patients with substantial increased risk for long-term mortality after kidney transplantation. Immun Ageing. (2020) 17:4. 10.1186/s12979-020-00175-z 32082402 PMC7020578

